# Dancing in local space: rolling hoop orbital amplification combined with local cascade nanozyme catalytic system to achieve ultra-sensitive detection of exosomal miRNA

**DOI:** 10.1186/s12951-022-01568-6

**Published:** 2022-08-02

**Authors:** Xin Gao, Haiping Wu, Yujian Li, Lu Zhang, Mingxuan Song, Xuhuai Fu, Rui Chen, Shijia Ding, Jiawei Zeng, Jia Li, Ping Liu

**Affiliations:** 1 Key Laboratory of Clinical Laboratory Diagnostics (Ministry of Education), College of Laboratory Medicine, Chongqing Medical University, Chongqing, 400016 People’s Republic of China; 2 Department of Orthopedics, The First Affiliated Hospital of Chongqing Medical University, Chongqing, 400016 People’s Republic of China; 3 Department of Clinical Laboratory, Chongqing University Cancer Hospital, Chongqing Cancer Institute, Chongqing Cancer Hospital, Chongqing, 400030 People’s Republic of China; 4 Department of Clinical Laboratory, School of Medicine, Mianyang Central Hospital, University of Electronic Science and Technology of China, Mianyang, 621000 People’s Republic of China; 5 The Center for Clinical Molecular Medical Detection, The First Affiliated Hospital of Chongqing Medical University, Chongqing, 400016 People’s Republic of China; 6 Bioscience (Tianjin) Diagnostic Technology CO., LTD, Tianjin, 300399 People’s Republic of China

**Keywords:** Rolling hoop orbital amplification, Local cascade nanozyme catalytic system, Exo-miRNA, ECL biosensor, Ultra-sensitive detection

## Abstract

The exosomal miRNA (exo-miRNA) derived from tumor cells contains rich biological information that can effectively aid in the early diagnosis of disease. However, the extremely low abundance imposes stringent requirements for accurate detection techniques. In this study, a novel, protease-free DNA amplification strategy, known as “Rolling Hoop Orbital Amplification” (RHOA), was initially developed based on the design concept of local reaction and inspired by the childhood game of rolling iron ring. Benefiting from the local space constructed by the DNA orbital, the circular DNA enzyme rolls directionally and interacts efficiently with the amplification element, making it nearly 3-fold more productive than conventional free-diffusion amplification. Similarly, the localized cascade nanozyme catalytic system formed by bridging DNA probes also exhibits outperformed than free ones. Therefore, a localized energized high-performance electrochemiluminescence (ECL) biosensor was constructed by bridging cascading nanozymes on the electrode surface through DNA probes generated by RHOA, with an impressive limit of detection (LOD) of 1.5 aM for the detection of exosomal miRNA15a-5p and a stable linearity over a wide concentration range from 10^− 2^ to 10^8^ fM. Thus, this work is a focused attempt at the localized reaction, which is expected to provide a reliable method for accurately detecting of exo-miRNAs.

**Graphical Abstract:**

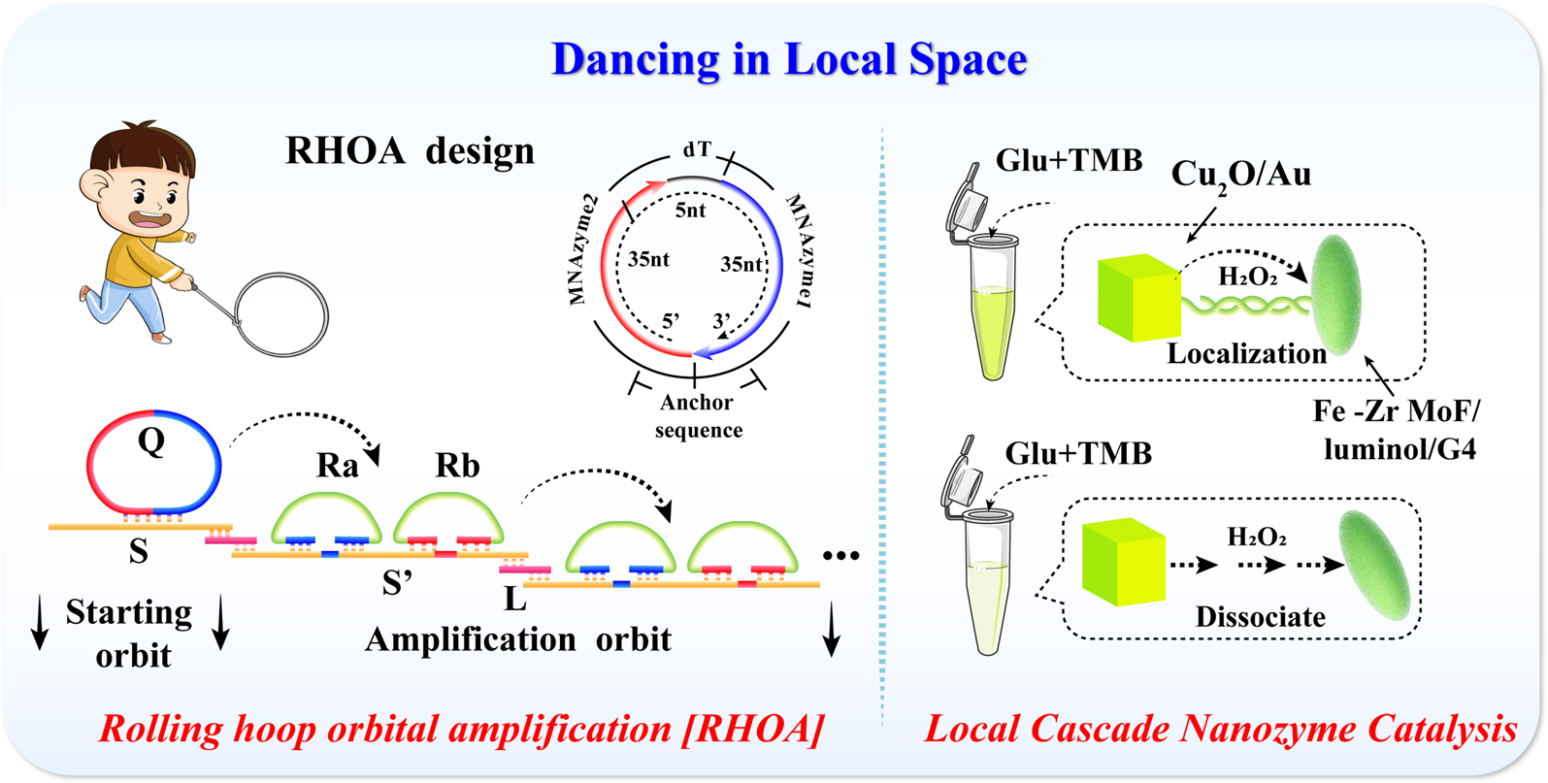

**Supplementary Information:**

The online version contains supplementary material available at 10.1186/s12951-022-01568-6.

## Introduction

Exosomes are small extracellular vesicles of 30–150 nm in diameter that are secreted by almost all cell types [1–3]. A variety of contents from parental cells, including mRNA, proteins and miRNAs are loaded into exosomes [4–7]. Among them, miRNA, a short (19–23 nucleotides), single-stranded non-coding RNA molecule with highly conserved functions, plays an irreplaceable role in mediating various regulatory pathways, such as organ development, cell proliferation, lipid metabolism, and viral replication [8, 9]. there are convincing evidences that aberrant expression of exo-miRNA is closely associated with a variety of malignant diseases, including cancer, heart disease and neurological disorders [10, 11]. Furthermore, exo-miRNA can be stably present in the peripheral circulation because of the protection of the exosomal lipid molecular layer [12–14]. Therefore, the detection of exo-miRNA can contribute to the early and precise diagnosis of diseases.

Nowadays, a diversity of techniques has been developed for exo-miRNA detection, including northern blotting [15], microarrays [15], and quantitative reverse transcription polymerase chain reaction (qRT-PCR) [16]. Although these techniques have been widely used in clinical, cumbersome operating steps, severe thermal cycles, and inevitable false positives are all nonnegligible limitations [17]. On the other hand, various biosensing technologies have been constructed as promising attempts for miRNA detection, such as a rapid electrochemical biosensor based on multifunctional DNA tetrahedron assisted catalytic hairpin assembly and surface-enhanced Raman scattering sensor based on plasmonic head-flocked gold nanopillars [18, 19]. In these explorations, electrochemical (EC)/ECL biosensing techniques are preferred by researchers because of their simplicity of operation, high sensitivity, and ease of miniaturization. Various EC/ECL biosensing strategies for the detection of exosomes and their derivatives have been constructed and have performed well for analysis [20–22]. However, EC/ECL biosensors have gradually revealed some limitations, such as insufficient sensitivity when directly applied to the detection of exo-miRNAs with low abundance, and the lack of high-performance EC signal probes [23–26]. Therefore, the construction of ultrasensitive EC/ECL biosensors for exo-miRNA remains a daunting challenge.

Enzyme-free nucleic acid isothermal amplification has attracted the attention of researchers as a novel amplification technique with simple operation, low cost and mild reaction conditions [27]. However, its lower amplification efficiency and limited sensitivity compared to enzyme-assisted amplification techniques have been an insurmountable challenge to overcome. The critical factors affecting the efficiency of non-enzymatic DNA amplification reactions are: (i) random diffusion of DNA substrates; (ii) variation in local probe concentration; and (iii) uncertain amplification routes. To overcome these deficiencies, the introduction of restrictive DNA spatial structures to confine the disorderly diffusion of reaction substrates has become a reliable way. For example, Jiang et al. utilized a tripod DNA scaffold to immobilize two metastable hairpins and reporter probes and confined them in a localized space to assemble a DNA nanomachine with high efficiency [28]; Qing et al. constructed a spatially positioned amplification reaction with accelerated target conversion to achieve sensitive detection of microRNA [29]. These attempts have fully verified the reliability of the localization reaction, but the complicated structure assembly and possible substrate leakage are issues that have to be considered in the strategic design. Moreover, simply putting together the existing enzyme-free amplification technology cannot break through the limitations of the existing technology, nor can it achieve a more meaningful breakthrough.

Furthermore, besides introducing localization reactions enhances the efficiency of non-enzymatic amplification reactions, the design of signal probes is another way to improve detection sensitivity. In interface platforms such as EC and ECL, immobilization of metal-nanozymes as electrocatalytic probes is already a mature strategy [30, 31]. Various metal-organic frameworks (MOF) [32, 33], composite nanoparticles [34] and other nanomaterials with peroxidase [35] or other biological enzyme activities [36] have been applied in the construction of biosensors. However, the catalytic activity of the metal nanozyme probe immobilized on the immobilization interface is significantly different from that in the bulk solution, due to the reduced conformational flexibility of the nanozyme. A promising solution lies in the possibility of immobilizing different types of enzymes in a precise sequence, sequentially designing a multi-step cascade reaction [37]. Fine spatial control can accelerate the reaction, reduce unnecessary side reactions, and decrease the accumulation of inhibitory or reactive intermediates [38, 39]. This is also the enablement of the localized response, and the multiple specificities of catalytic substrates in the cascade enzyme system can effectively avoid the non-specific signal catalyzed by a single enzyme probe and improve the signal-to-noise ratio of detection [40].

To eliminate the drawbacks of the enzyme-free signal amplification reaction, and broaden the technical approach for detection of exo-miRNA, driven by the concept of localized reaction, a novel and protease-free DNA amplification strategy, named “Rolling Hoop Orbital Amplification” (RHOA), has been developed and combined with cascade nanozyme catalyzed reaction to construct an ECL biosensor. The RHOA is inspired by the hoop-rolling game played in childhood and the directional rolling path of circular DNA can be formed by simply splicing the starting orbit and the amplified orbit. With the addition of the target exo-miRNA, the pried circular DNA enzyme achieves localized targeted rolling cleavage through a foothold-mediated strand substitution reaction, thereby releasing the signal probe for efficient and orderly signal amplification. On the surface of ECL electrode, Fe-Zr MOF/G4 nanozyme with peroxidase activity [41, 42] and Cu_2_O/Au with glucose oxidase activity [43, 44] form a cascade catalytic reaction in a local space through the signal probe. In the presence of low-dose glucose, the cascade nanozyme system can catalyze luminol immobilized in Fe-Zr MOF to produce a high ECL signal. Under the cogitation of localized reaction, the entire reaction system has the advantages of simplicity, efficiency, high sensitivity, and high specificity, which satisfies the current clinical performance requirements for exo-miRNA detection technology and is expected to open up an achievable technical approach for ultra-sensitive miRNA detection.

## Experimental

### Reagents and materials

Luminol (98%), chloroauric acid hexahydrate (HAuCl_4_·6H_2_O), chloroplatinic acid hexahydrate (H_2_PtCl_6_·6H_2_O), zirconium oxychloride octahydrate (ZrOCl_2_·8H_2_O), benzoic acid, ascorbic acid, cupric sulfate (CuSO_4_), sodium hydroxide (NaOH), polyvinylpyrrolidone (PVP, MW = 40,000 g/mol), ammonium persulfate (APS, 98.5%), 6-Mercapto-1-hexanol (MCH), sodium borohydride (NaBH_4_) and Chitosan were all supplied from Sigma-Aldrich (St. Louis, MO, USA). *N*,*N*-dimethylformamide (DMF, 99.8%), hydrogen peroxide (H_2_O_2_, 20%), and ethanol were purchased from Sangon Inc. (Shanghai, China). Sulfuric acid (H_2_SO_4_) was purchased from Kelong Chemical Inc. (Chengdu, China). Fe (III) tetra (4-carboxyphenyl) porphine chloride (TCPP (Fe), 97%) was bought from Frontier Scientific (Logan, Utah, USA). 3,3′,5,5′-Tetramethylbenzidine (TMB) was purchased from Beyotime Institute of Biotechnology (Shanghai, China). All oligonucleotides purified by high-performance liquid chromatography were obtained from Sangon Inc. (Shanghai, China) and listed in Additional file 1: Table S1. All clinical samples were obtained from the Chongqing Cancer Hospital, and the informed consent of patients and the permission of the Medical Ethics Committee were obtained. Other reagents were of analytical grade and without further purification. The experimental systems involving miRNA participation in this work all used DEPC-containing deionized water. Other experiments used deionized water (≥ 18 MΩ cm, Milli-Q, Millipore) from the Millipore water purification system.

### Apparatus

ECL measurements were monitored and recorded by MPI-E capillary electrophoresis electrochemiluminescence detector (Xi’an Remax Analysis Instruments Co. Ltd., China) and electrochemical impedance spectroscopy (EIS) and cyclic voltammetry (CV) were carried out with CHI660D electrochemical workstation (Shanghai Chenhua Instruments Co. Ltd., China). A conventional three-electrode system was utilized with a platinum wire as auxiliary electrode, Ag/AgCl as the reference electrode, and modified glassy carbon electrode (GCE, Ф = 4 mm) as the working electrode during ECL detection. Morphology and elemental mapping analysis of different nanomaterials were characterized by the scanning electron microscope (SEM, Hitachi, SU-8020, Tokyo, Japan) and transmission electron microscope (TEM, FEI Company, Tecnai G2 F20, USA). Transmission electron microscope (TEM, JEOL, JEM 1200EX, Japan) and Nanoparticle Tracking Analysis (NTA, Particle Metrix, ZetaView PMX 110, Germany) was selected to characterized the morphology and size of exosomes. Atomic force microscopy (AFM) for RHOA characterization was performed by SPM9700HT AFM (Shimadzu, Japan). In addition, the absorption spectrum of the nanomaterial is recorded by Ultraviolet-visible (UV-vis) spectrophotometer (Shimadzu, UV-2450, Kyoto, Japan). Fluorescent spectra were measured using Agilent Technologies (Palo Alto, CA, USA). Native polyacrylamide gel electrophoresis (PAGE) relied on Bio-Rad (USA).

### Preparation of buffers

5 × Tris-HCl buffer (pH 7.4): 100 mM NaCl, 25 mM KCl, 50 mM Tris; 1 × TE buffer (pH 8.0): 20 mM NaCl, 5 mM KCl, 10 mM Tris, 1 mM EDTA; 10 × PBS buffer (pH 9.0): NaCl 40 g, KCl 1 g, Na_2_HPO_4_ 7.2 g, KH_2_PO_4_ 1.3 g, 500 mL deionized water; 5 × TBE buffer (pH 8.0): 445 mM Tris, 445 mM boric acid, 10 mM EDTA; TMA buffer (pH 7.4): 80 mM NaCl, 20 mM KCl, 40 mM Tris, 20 mM acetic acid, 2 mM EDTA, 10 mM MgCl_2_.

### Construction of RHOA

The RHOA was divided into two parts, starting orbit and amplification orbit respectively and connected by the L strand in the middle. Starting orbit: the synthesis and characterization of circular DNA Q were shown in Additional file 1: Fig. S1. Orbit strand “S” and circular DNA “Q” were mixed at a concentration ratio of 1:0.6, where the concentration of S was 500 nM, and incubated at 37 °C for 1 h to obtain the starting orbit. Amplification orbit: the orbit strand S’ and the probes Ra and Rb were mixed at a concentration ratio of 4:2:2, where the concentration of S’ was 1 µM. The mixture was heated at 95 °C for 5 min and then slowly dropped to room temperature to assemble the amplification orbit. Assembly of RHOA structure: the products of starting orbit and amplification orbit were mixed with L chain, so that the concentration ratio of S, S’ and L chain was 1:2:2. The mixture was incubated at 37 °C for 1 h to obtain the complete RHOA structure.

### Preparation of Cu_2_O/Au nanocube

The Cu_2_O/AuNPs nanocube synthesized in this work referred to a previous report [45]. 0.16 g CuSO_4_ and 0.2 g PVP were dissolved in 100 mL of deionized water and stirred at room temperature for 1 h. Then, NaOH solution (1.5 M, 25 mL) and ascorbic acid solution (0.1 M, 25 mL) were successively added drop by drop. It could be observed that the blue precipitate was initially produced and gradually changed to brick red. After washing, vacuum drying, and weighing, 5 mg Cu_2_O was ultrasonically dispersed in 30 mL deionized water, and then HAuCl_4_ solution (1%, 50 µL) was injected while maintaining ultrasonic. Finally, the pellet was centrifuged, washed with deionized water, and stored at 4 °C in the dark.

### Synthesis of Fe-Zr MOF/luminol/G4/T

Fe-Zr MOF was synthesized according to the reported method with slight modification [46, 47]. First, ZrOCl_2_·8H_2_O (300 mg), TCPP(Fe) (100 mg) and benzoic acid (2.2 g) were ultrasonically dissolved in 100 mL DMF solution. Then, the mixture was stirred and heated to 95 ℃ for 5 h. After the reaction solution cooled to room temperature, the formed purple-red Fe-Zr MOF was collected by centrifugation at 15,000 rpm for 15 min, and washed twice with DMF. Finally, the products were re-dispersed in DMF for storage.

20 mL Fe-Zr MOF was mixed with 15 mL luminol (0.1 M) and stirred at room temperature for 10 h, then washed twice with deionized water by centrifugation and redispersed in 20 mL water. Finally, 200 µL 100 µM G4 DNA strand and T strand were added to the above solution and left standing at 4 ℃ for 12 h. Fe-Zr MOF /luminol/G4/T was obtained after being washed twice.

### Assembly of ECL biosensor and detection of exo-miRNA

The bare GCE was polished with 0.3 and 0.05 μm alumina (Al_2_O_3_) slurry for 3 min to obtain a mirror-like surface, then thoroughly ultrasonically cleaned in deionized water and dried with nitrogen. Cu_2_O/Au nanocubes are dropped onto the electrode surface firstly and dried at 37 °C. Subsequently, 10 µL P chain (100 nM) was added dropwise to the electrode, incubated overnight at 4 °C, and then washed with 1 × Tris-HCl buffer to remove unbound P. Then, the RHOA products triggered by different concentrations of exo-miRNA-15a-5p and the Fe-Zr MOF /luminol/G4/T probe were incubated on the electrode at 37 °C for 1 h at the same time. Finally, the constructed electrode was washed and soaked in 10×PBS buffer containing 100 mM glucose to record ECL signal.

The experimental procedures such as the extraction of exosomes and miRNA are described in detail in Additional file 1: S1–S6.

## Results and discussion

### Design principle of the ECL biosensor

**Scheme 1:**
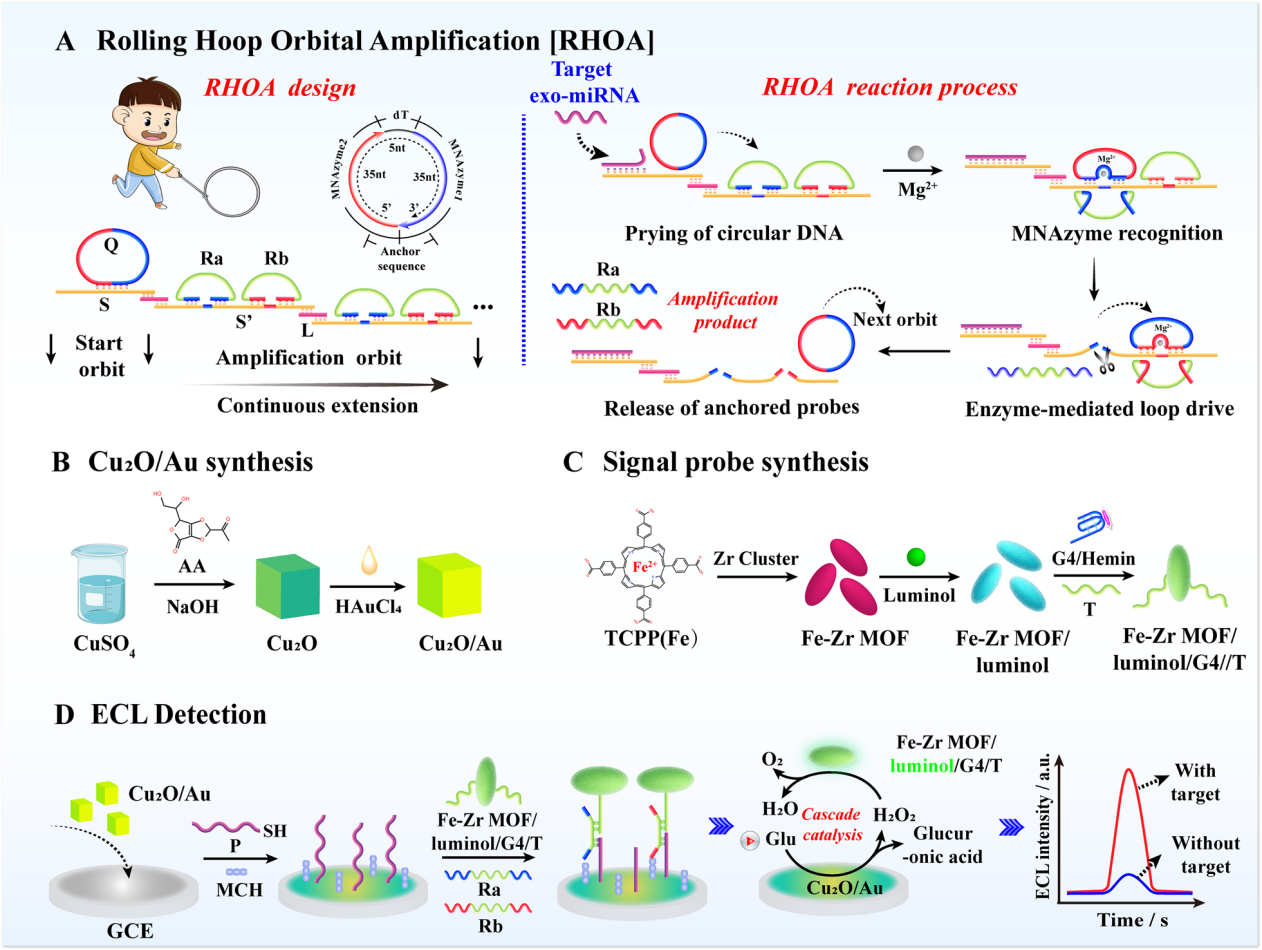
Schematic illustration of the construction of developed ECL biosensor based on RHOA and local cascade nanozyme catalysis system. **A** The structure design of RHOA and the reaction process of miRNA triggering RHOA. **B** The synthesis step of Cu_2_O/Au. **C** The synthesis step of Fe-Zr MOF/luminol/G4/T. **D** The electrode surface assembly of ECL biosensor

Scheme 1 illustrates the developed ECL biosensor based on the RHOA and cascade metal nanozyme catalytic system. The detailed design of RHOA is shown in Scheme 1A. Inspired by the hoop-rolling game played in childhood, the main structure of RHOA is divided into circular DNA, starting orbit, and amplification orbit. In the design of circular DNA, two MNAzyme sequences activated by Mg^2+^ are introduced, named MNAzyme 1 and MNAzyme 2, and they are anchored on the starting orbit by the principle of base complementary pairing. The starting orbit is connected with the continuously extending downstream amplification orbit through a bridge DNA “L”. Two signal probes, Ra and Rb, are arranged on the downstream amplification orbit in an orderly manner, which have the same functional sequence and different anchor sequences.

In this study, exo-miRNA-15a-5p was selected as the detection target because of its specific and high expression in endometrial cancer, a malignant disease with a high incidence of women. After miRNA-15a-5p is added to the RHOA reaction system, the anchored circular DNA is pried due to strand displacement, just like a naughty child pushing the iron ring with a push rod. With the precise base design, the MNAzyme 1 sequence of circular DNA first intervened into the double-strand of Ra and amplifies orbit by a toehold, replacing the free single-strand of Ra. Subsequently, MNAzyme 1 activates the cleavage activity in the presence of Mg^2+^, cuts the amplified orbit and detaches from the anchor position. The other half of the circular DNA sequence MNAzyme 2 continues to replace the corresponding Rb probe in the same way. The continuous displacement-cut reaction and sequence restriction drive the directional rolling of circular DNA to achieve high-efficiency signal amplification in the local area, and generate a large number of Ra and Rb probes.

Another important part of the ECL biosensor is shown in Scheme 1B/C. Cu_2_O/Au is synthesized by reducing Au atoms in situ on the surface of Cu_2_O. The incorporation of Au not only enhances the glucose oxidase activity of Cu_2_O, but also provides active sites for DNA connection. Fe-Zr MOF is an oblong nanomaterial with peroxidase activity that has been proven. The large specific surface area provides abundant sites for the immobilized ECL substrate luminol, and the incorporation of Zr nanoclusters provides stable anchor points for introducing phosphoric acid group-modified G4 nanozyme, which further enhances the catalytic ability of Fe-Zr MOF.

The detection process on the electrode surface is shown in Scheme 1D. The functional sequence region of the Ra and Rb generated by RHOA connects the Fe-Zr MOF/luminol/G4 probe to the electrode surface deposited with Cu_2_O/Au. After glucose is added, Cu_2_O/Au exerts glucose oxidase activity, generates a large amount of H_2_O_2_ in a local space. Fe-Zr MOF/G4 catalyzes hydrogen peroxide to form oxygen free radicals to act on the luminol, thereby obtaining strong ECL signal and realizing ultra-sensitive detection of exo-miRNA.

### Verification of RHOA

**Fig. 1:**
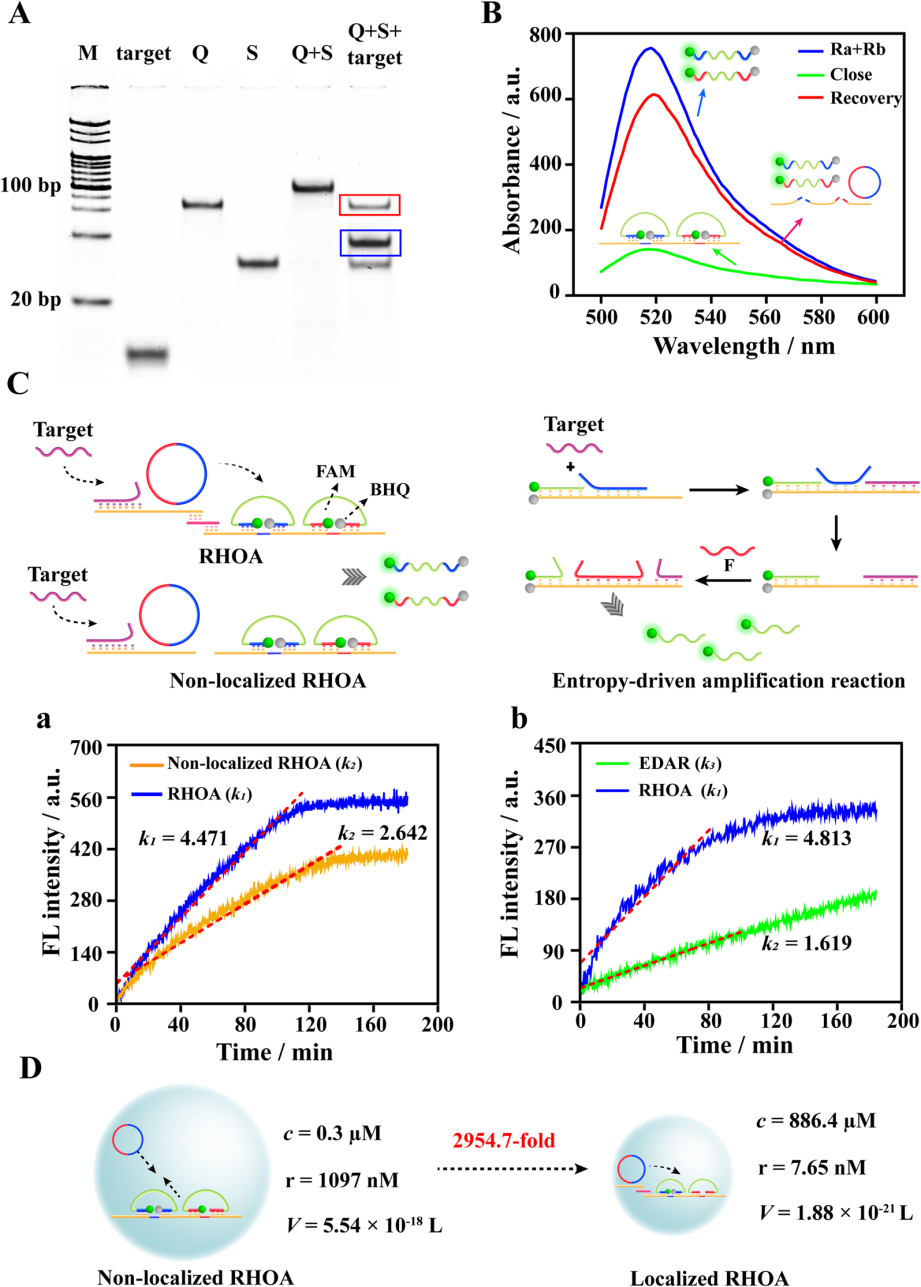
**A** Characterization of starting orbit via PAGE. The concentration of target and S was 1 µM. The concentration of Q was 500 nM. **B** Characterization of amplification orbit via fluorescence. The concentration of Ra and Rb probe was 50 nM. The concentration of S’ was 100 nM. **C** Comparison of RHOA amplification efficiency. Schematic Illustration of the amplification process of RHOA, non-local RHOA and the classic enzyme-free DNA amplification technology-entropy-driven amplification reaction (EDAR). **a** Fluorescence kinetic curves of RHOA and non-local RHOA, *k*_*1*_: the amplification rate of RHOA, *k*_*2*_: the rate of non-local RHOA. **b** Fluorescence kinetic curves of RHOA and EDAR, *k*_*1*_: the amplification rate of RHOA, *k*_*3*_: the rate of EDAR. **D** Schematic drawing of comparison of the reaction area and local concentration of localized RHOA and non- localized RHOA

The feasibility of RHOA was verified by 12% PAGE, fluorescence and AFM. As shown in Fig. 1A, Circular DNA “Q” (The synthesis of Q was shown in Additional file 1: Fig. S1) and orbit DNA “S” assembled to form the initial starting orbit (lane 5). In the present of miRNA-15a-5p, free “Q” (red frame) and the hybridization product of miRNA and “S” (blue frame) appeared in lane 6, which indicated that miRNA-15a-5p could be recognized and captured by the orbit and segregated the circular DNA “Q”. The above results all illustrated that the assembly of the starting orbit was successful, and miRNA-15a-5p could pry the “Q” to initiate the amplification reaction. On the other hand, the two ends of the Ra and Rb probes were modified with the fluorescent group -FAM and the quenching group -BHQ, respectively. The assembly of the amplified orbit was verified by detecting the fluorescence intensity. It could be seen from Fig. 1B that compared with the free Ra and Rb probes, the fluorescence intensity of the assembled amplification orbit decreased significantly. With the addition of Q, the RHOA reaction initiated directly, and the fluorescence intensity was recovered by about 80%. The AFM characterization results were shown in Additional file 1: Fig. S2. it could be clearly observed that distinct long DNA strands formed with the assemble of starting and amplification orbits (Additional file 1: Fig. S2A). After the RHOA reaction, the long DNA strands were digested by MNAzyme cleavage (Additional file 1: Fig. S2B). All the above results showed that RHOA was feasible and stable.

The feasibility and amplification efficiency of the entire RHOA reaction system are more critical. First, the amplification efficiency of localized and non-localized RHOA reaction was analyzed by fluorescence kinetics. The detailed design principle was shown in Fig. 1C. It was not difficult to see from Fig. 1Ca that, compared to the free starting and amplification orbits, the localized RHOA reaction formed by “L” stand coupling performed a higher reaction rate. *K*_*1(RHOA)*_ was about 1.7 times that of *K*_*2 (Non-localized RHOA)*_. The comparison between RHOA and the classic enzyme-free DNA amplification reaction, *Entropy Driven Amplification Reaction* (EDAR), was more obvious (Fig. 1Cb). The localized design and orderly orbital amplification endowed RHOA with excellent detection performance. *K*_*1(RHOA)*_ was about 3 times that of *K*_*3(EDAR)*_. The above results fully illustrated that the localized spatial design might promote a higher local concentration of the amplification element and more rapid interaction, thereby greatly improving the amplification efficiency.

To further elucidate the mechanism by which localized design improves the efficiency of enzyme-free amplification reactions, collision frequency theory was introduced to explore the amplification process. As shown in Fig. 1D, according to the Avogadro’s Hypothesis: *V* = 1/*c*N and the sphere volume formula: *V* = 4πr^3^/3, the local concentration of localized RHOA and non-localized RHOA was calculated, in which *V* is the volume of local sphere, *c* is the concentration of starting and amplification orbits (0.3 µM in this experiment), N is the Asgardro constant (6.02 × 10^23^), and r is the radius of sphere. For localized RHOA, the distance between Q and Ra was designed to be 45 nt and the length was approximately 15.3 nm. According to the above equation, the concentration of the local starting and amplification orbits could reach to 886.4 µM, which was 2954.7 folds higher than that of non-localized RHOA. The local high concentration avoided the long two-way travel between Q and Ra/Rb, greatly improved the amplification efficiency, and manifested as significantly accelerated reaction kinetics.

### Characterization and verification of cascade nanozyme system

**Fig. 2:**
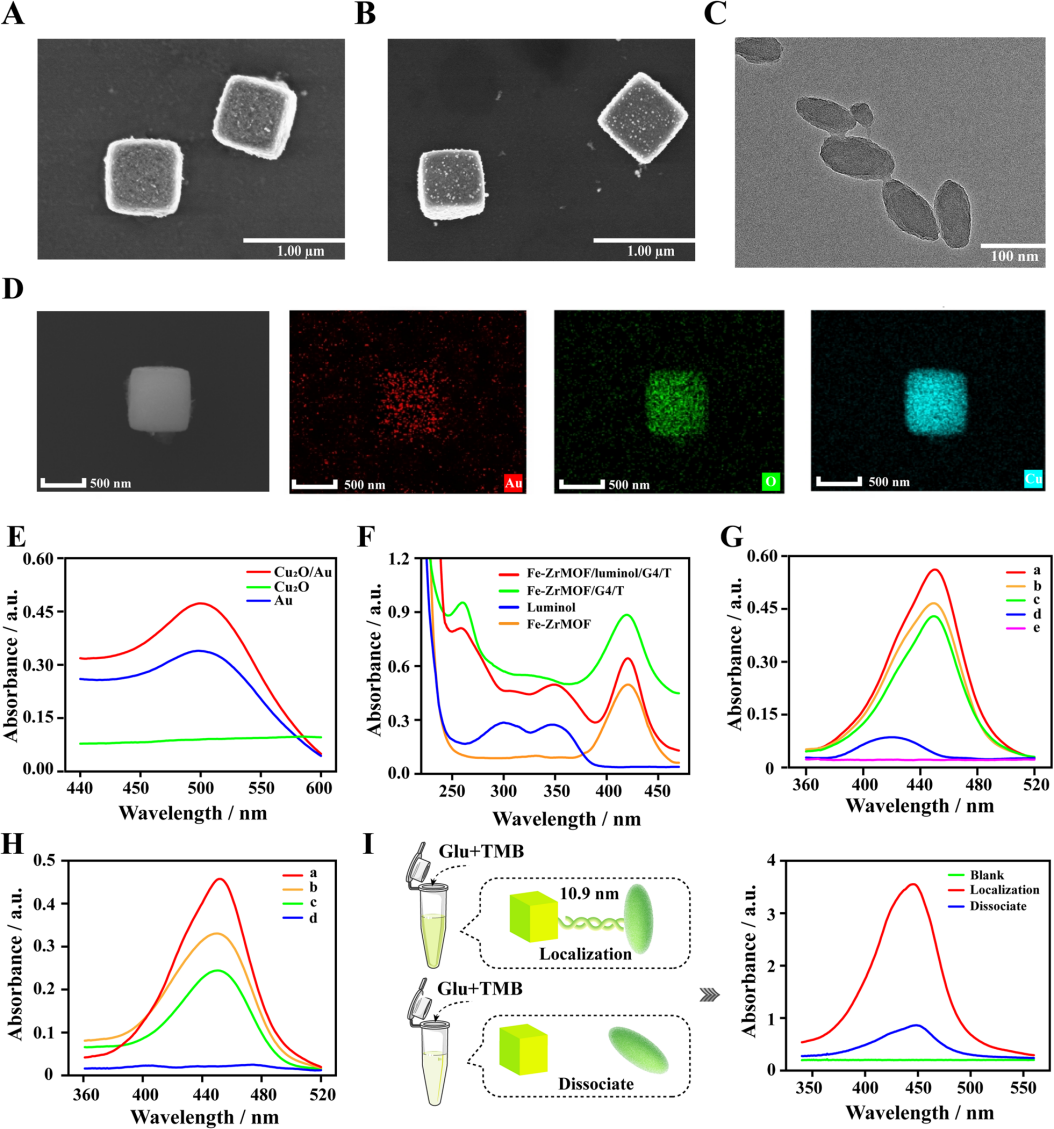
SEM images of Cu_2_O (**A**) and Cu_2_O/Au (**B**). **C **TEM image of Fe-Zr MOF. **D** EDS-mapping images of Cu_2_O/Au. **E** UV characterization of Cu_2_O/Au. **F** UV characterization of Fe-Zr MOF /luminol/G4/T. **G** Verification of the peroxidase catalytic performance of Fe-Zr MOF/G4. Fe-Zr MOF/G4 + TMB + H_2_O_2_ a), Fe-Zr MOF + TMB + H_2_O_2_ b), G4 + TMB + H_2_O_2_ c), Fe-Zr MOF + Glu + TMB d) and TMB + H_2_O_2_ e). **H** Verification of the glucose oxidase catalytic performance of Cu_2_O/Au. Cu_2_O/Au + Glu + Fe-Zr MOF /G4 + TMB a), Cu_2_O + Glu + Fe-Zr MOF /G4 + TMB b), Au + Glu + Fe-Zr MOF /G4 + TMB c) and Cu_2_O/Au + Glu + TMB d). **I** The local and non-local cascade nanozyme catalytic system reaction mode and colorimetric experiment results

The size and morphology of Cu_2_O/Au were characterized by SEM. Compare with pure Cu_2_O (Fig. 2A), Cu_2_O/Au (Fig. 2B) could be observed to have the same cube shape and ~ 500 nm size. The difference was that the surface of Cu_2_O/Au was deposited by uniform Au nanoparticles, which was consistent with the results of SEM-EDS (Fig. 2D). UV absorption spectroscopy was also used to verify the synthesis of Cu_2_O/Au. As shown in Fig. 2E, Cu_2_O/Au possessed an obvious absorption peak at 520 nm, which was the characteristic absorption peak of Au NPs. The above results illustrated the successfully synthesis of Cu_2_O/Au. In addition, the stability of the aqueous Cu_2_O solution was verified by observing the oxidation of the Cu_2_O solution at different storage time. It could be clearly contrasted that no significant oxidation of Cu_2_O solution was occurred within 14 days, indicating its stable preservation (Additional file 1: Fig. S3).

Another nanozyme- Fe-Zr MOF/Luminol/G4/T was characterized by TEM, XRD, BET and UV. As shown in Fig. 2C, Fe-Zr MOF exhibited an elliptical structure with a size of about 100 nm. the XRD results also remained consistent with the previous studies, indicating the successful synthesis of Fe-Zr MOF [48–50] (Additional file 1: Fig. S4). Furthermore, the Brunauer-Emmett-Teller (BET) data clearly demonstrated that the surface area and pore space of Fe-Zr MOF were 775.9996 m²/g and 2.4776 nm, respectively, which could achieve a better loading of DNA probes (Additional file 1: Fig. S5). Finally, through UV analysis, 420 nm was confirmed to be the characteristic absorption peak of Fe-Zr MOF, while 294 nm and 351 were of luminol, and 260 was of DNA. These characteristic peaks could all be observed in the UV absorption spectrum of Fe-Zr MOF/luminol/G4/T (Fig. 2F). Thus, Fe-Zr MOF/luminol/G4/T was been proved to be successfully constructed.

As reported in previous studies, both Fe-Zr MOF and G4 have the peroxidase-like activity. In addition, our research group has also explored the catalytic performance of different G4 nanozyme structures in previous reports [35, 42, 51]. In this study, we also verified the catalytic capabilities of different G4 structures (Additional file 1: Fig. S6). As shown in Fig. 2G, compared with the control Fe-Zr MOF and G4 nanozyme, Fe-Zr MOF/G4 expressed higher activity in the H_2_O_2_ catalytic system with TMB as the substrate. Besides, the glucose oxidase activity of Cu_2_O/Au was also verified by colorimetric experiments. The performance of individual Cu_2_O and Au nanoparticles after being cascaded with Fe-Zr MOF/G4 was lower than that of Cu_2_O/Au (Fig. 2H). Therefore, through a series of comparative experiments, it was confirmed that Fe-Zr MOF/G4 and Cu_2_O/Au possessed excellent peroxidase and glucose oxidase catalytic ability, respectively, and the feasibility of the cascade enzyme system was also confirmed. Finally, the local cascade nanozyme catalytic system constructed by DNA ligation had more efficient catalytic performance than the free cascade nanozyme (Fig. 2I). The improvement in catalytic performance might benefit from refined local space control, which could effectively increase the concentration of the co-reactant in the catalytic space, thereby accelerating the catalytic reaction, reducing unwanted side reactions, and reducing the accumulation of reactive intermediates.

### Feasibility of the ECL biosensor

**Fig. 3:**
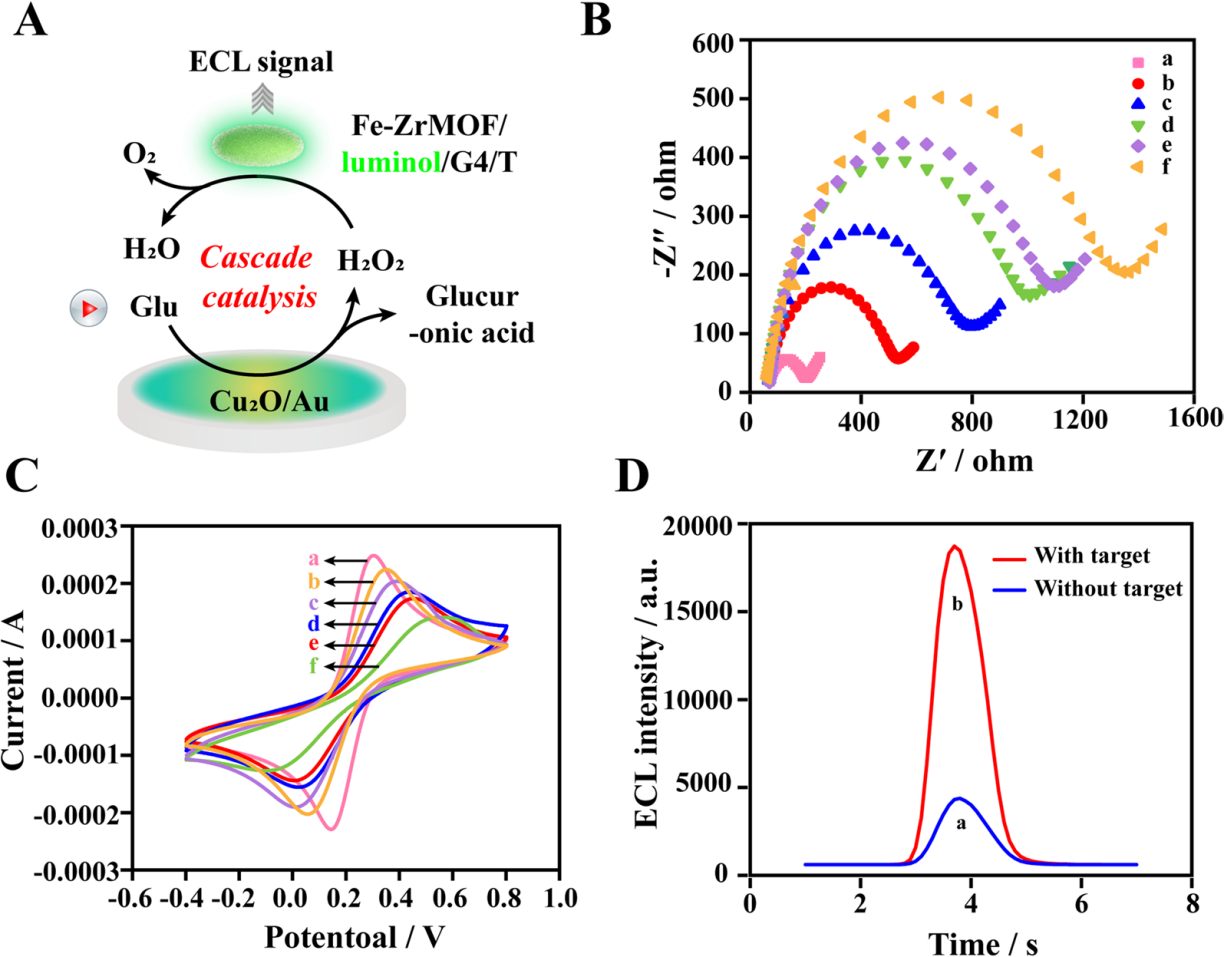
Feasibility of developed ECL biosensor. **A** The ECL biosensor signal generation pathway. **B** EIS and (**C**) CV of different modified electrodes in 5 mM [Fe(CN)_6_]^3−^/^4−^ solution containing 0.1 M KCl: (a) bare electrode; (b) Cu_2_O/Au; (c) P probe; (d) MCH; (e) RHOA products; (f) Fe-Zr MOF /luminol/G4/T.** D** The ECL signal for constructed biosensor in the detection solution containing 100 mM glucose without (a) and with (b) exo-miRNA-15a-5p

The ECL biosensor relied on the signal probes generated by RHOA to form a local cascade enzyme catalytic system on the electrode surface (Fig. 3A), and glucose were serviced as the catalytic substrate to initiate the ECL signal generation pathway. In ordered to confirm the progressive assembly process of the ECL biosensor, electrochemical impedance spectroscopy (EIS) and cyclic voltammetry (CV) measurements were performed in 5 mM [Fe(CN)_6_]^3−^/^4−^ solution containing 0.1 M KCl. In EIS, the curve with a semicircular diameter was equal to the electron transfer resistance (Ret). As shown in Fig. 3B, the bare glassy carbon electrode existed a small resistance. With the deposition of Cu_2_O/Au, the resistance of the electrode surface increased greatly, which was due to the weaker conductivity of Cu_2_O/Au. The addition of probe P and MCH lead to a continuous increase of resistance, indicating the successful modification. After RHOA amplification, miRNA-15a-5p could generated a large amount of Ra and Rb. Curve e showed the resistance when the amplification system was incubated on electrode. It could be observed that the radius was widened compared to curve d. Finally, the Fe-Zr MOF /luminol/G4 modified by the probe T was added, and the functional sequence of Ra and Rb connected the probe P and the probe T together. The resistance reaches the maximum value, indicating the final formation of the three-layer complex. Figure 3C showed that the CV results at different stages were in good agreement with those measured by EIS. Moreover, the ECL signal shown in Fig. 3D illustrated only the presence of target exo-miRNA-15a-5p could produce an obvious signal with nearly 5 times the signal-to-noise ratio. The above results fully demonstrate that the developed ECL sensor was feasible and reliable.

### Optimization of the ECL biosensor

Several experimental conditions were optimized to obtain an optimal analytical performance of the ECL biosensor, such as the concentration ratio of Q and S, the concentration ratio of Ra, Rb and S’, the reaction time of RHOA and the hybridization time of probes on the electrode surface. The starting orbit and amplification orbit are the mainly components of RHOA. Excessive or insufficient concentrations of circular DNA “Q” and amplified products “Ra” and “Rb” probes may have an immeasurable impact on the amplification efficiency of RHOA. In particular, the leakage of Q, Ra or Rb would cause high background signal. Thus, a series of concentration ratios of Q and S, and, Ra, Rb and S’ was set to evaluate amplification performance. As shown in Additional file 1: Fig. S7A/B, the SNR of the ECL biosensor reached the peak when the concentration of Q and S was 0.6:1 and the concentration of Ra, Rb and S’ was 2:2:4. Another key condition of the ECL biosensor was the reaction time. First, various time gradient was selected to detect the RHOA analysis performance. The results illustrated in Additional file 1: Fig. S7C clearly showed that the ECL signal rose with time, while the background signal also slowly increases. Thus, 90 min was selected as the optimal reaction time of RHOA according to the SNR. Similarly, ECL signal was collected at different hybridization time points of probes on the electrode surface (Additional file 1: Fig. S7D). At 60 min, the signal-to-noise ratio reached its peak and then entered a plateau. Therefore, 60 min was the optimal probe hybridization time. Other experimental conditions were also optimized and the results were shown in Additional file 1: Fig. S8–S10.

### Sensitivity of the ECL biosensor

**Fig. 4:**
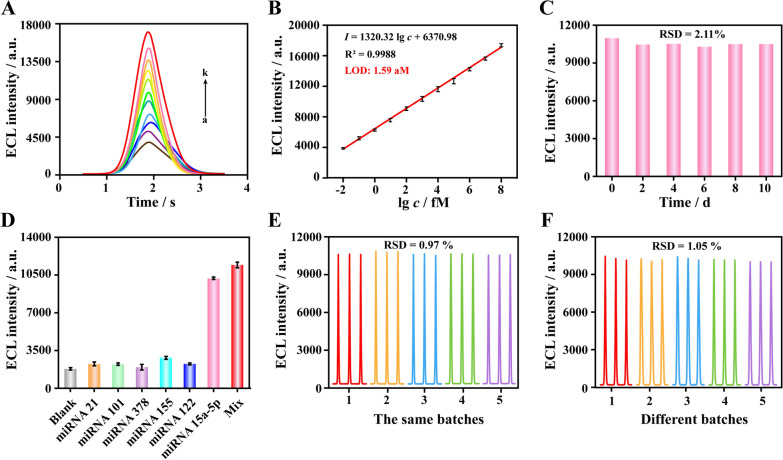
Evaluation of the analyze performance of the ECL biosensor. **A** ECL signal responding to 10^− 2^, 10^− 1^, 1, 10, 10^2^, 10^3^, 10^4^, 10^5^, 10^6^, 10^7^, 10^8^ fM of exo-miRNA-15a-5p (from a to k). **B** Linear correlation between the ECL intensity and the logarithm of target concentrations ranging from 10^− 2^ fM to 10^8^ fM. **C** Evaluation of the stability of the ECL biosensor. **D** Verification of the specificity of the ECL biosensor. The current ECL signal respond to blank, miRNA-21, miRNA-101, miRNA-378, miRNA-155, miRNA-122, miRNA-15a-5p and mixture. **E** Evaluation of the intra-batch repeatability of purposed ECL biosensor. **F** Evaluation of the inter-batch repeatability of purposed ECL biosensor. All results expressed as mean ± standard variation (n = 3)

The concentration of miRNA in exosomes is extremely low, which puts forward almost harsh requirements on the sensitivity of exo-miRNA detection technologies. Under the optimal experimental conditions, the sensitivity of prepared ECL biosensor was explored. Various ECL signal was obtained under a series of miRNA-15a-5p concentration gradient. After linear regression analysis, it was clearly seen that ECL intensity continued to increase as the concentration of miRNA-15a-5p increased, and within the range of 10^−2^–10^8^ fM, there was a good linear relationship between ECL signal and the logarithmic value of concentration. The corresponding linear regression equation was *I* = 1320.32 lg*c* + 6370.98 (*I*: ECL signal, *c*: the concentration of exo-miRNA-15a-5p), and the correlation coefficient (*R*^*2*^) was 0.9988 (Fig. 4A, B). According to the 3σ rule, the limit of detection (LOD) of miRNA-15a-5p was calculated to be 1.59 aM, which was far beyond the numerous miRNA detection technologies in previous studies (Additional file 1: Table S2). The excellent detection sensitivity benefitted from the localized RHOA and the cascade nanozyme system with high catalytic performance. The breakthrough in sensitivity also laid the foundation for the ECL biosensor to be applied for actual exo-miRNA detection.

### Stability, specificity, repeatability and reproducibility of the ECL biosensor

In order to assess long-term stability of the ECL biosensor, the prepared electrode was stored at 4 ℃ and examined signal response every two days. On the tenth day, the signal ECL still reached to 95.83% of the initial value, and the RSD of five detection results was only 2.11%, which fully proved that the developed ECL biosensor was capable of long-term preservation (Fig. 4C).

Furthermore, various exo-miRNAs, such as miRNA-21, miRNA-101, miRNA-378, miRNA-155 and miRNA-122, were selected as interferences and added to the ECL biosensing system to assess specificity. As shown in Fig. 4D, only the mixed substrate and miRNA-15a-5p obtained obvious ECL signal, where the concentration of miRNA-15a-5p was only 1 pM, and the signal generated by other 100 nM interference was almost the same as the blank. These results indicated that the ECL biosensor possessed reliable specificity.

The intra- and inter-batch repeatability of the ECL biosensor was also evaluated. As shown in Fig. 4E, the signal response generated by 1 pM target was collected from 5 ECL biosensors respectively, which prepared in the same batch under the same conditions. The results showed that the intra-group RSD was 0.97%. Similarly, 5 ECL biosensors from different batches examined the same concentration of target, showing an inter-batch variation of only 1.05% (Fig. 4F). Further, different operators performed the assay for the same concentration of target, thus verifying the reproducibility of the ECL biosensor. As shown in Additional file 1: Fig. S11, the RSD value of the assay results for five operators was 1.06%. There was no doubt that the purposed ECL biosensor possessed excellent detection repeatability and reproducibility, which guaranteed the reliability of results.

### Analysis of exo-miRNA in clinical samples

Realizing the detection of exo-miRNA-15a-5p in actual sample matrix is a necessary approach for the ECL biosensor to move towards clinical application. Therefore, serum exosomes were extracted and characterized firstly (Additional file 1: Fig. S6), and three different concentrations of miRNA-15a-5p (10^7^, 10^3^, 10^− 1^ fM) were added to the exosome lysate for recovery experiments. The detection results in Table 1 showed that the ECL biosensor had an acceptable recovery rate of 99.7-104.0%, with RSD between 1.06 and 1.21%, which demonstrated the ability to resist complex matrix interference and the potential for clinical application.

**Table 1:** Assay results of exo-miRNA-15a-5p spiked into the exosome lysate using the developed ECL biosensor

Sample	Addition [fM]	ECL Intensity [a.u.]	Found [fM]	RSD [%, n = 3]	Recovery [%, n = 3]
1	10^7^	15611.33	9967815.71	1.21	99.7
2	10^3^	10342.33	1018.12	1.19	101.8
3	10^− 1^	5078.66	0.10	1.06	104.0

**Fig. 5:**
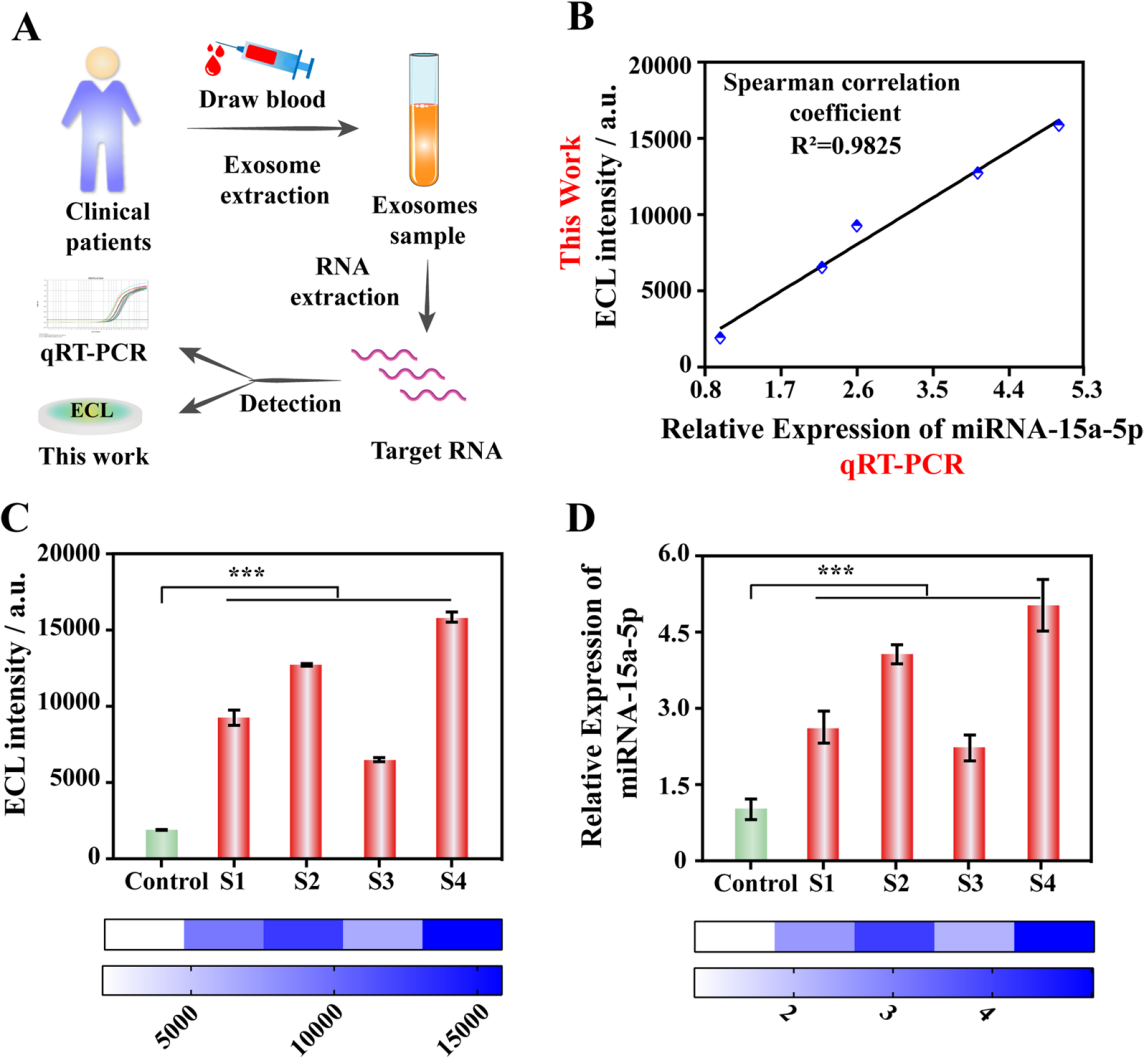
Analysis of clinical sample detection results. **A** Schematic drawing of the clinical sample testing process. **B** Correlation analysis between the detection results of ECL biosensor and qRT-PCR. **C**, **D** Are the detection results of miRNA-15a-5p in serum exosomes (S1–S4) of clinical endometrial cancer patients detected by the ECL biosensor and qRT-PCR, respectively. The “Control” group selected serum exosomes in healthy people

Furthermore, clinical sample testing was carried out to evaluate the practical application performance of the ECL biosensor. Serum exo-miRNAs from endometrial cancer patients were extracted as detection samples, and the detection results were compared with those of qRT-PCR. As shown in Fig. 5, compared with control sample from healthy people, the content of miRNA-15a-5p in serum exosomes of endometrial cancer patients was remarkably increased, with a significant statistical difference, and the results of qRT-PCR also verified this trend. Moreover, the correlation analysis between the detection results of the ECL biosensor and qRT-PCR showed that the Poisson correlation coefficient reached to 0.9825, with excellent consistency. Therefore, the above results fully demonstrated that the developed ECL biosensor could accurately detect low-abundance miRNA in serum exosomes, and has the potential to achieve clinical translational application.

## Conclusions

Accurate analysis of exo-miRNA is still an arduous challenge, which is worth the persistent efforts of researchers. In this study, from the perspective of localized reaction, a novel enzyme-free DNA amplification strategy, named RHOA, was developed, and coupled with localized cascade nanozyme catalytic system, a proposed ECL biosensor was constructed to achieve ultra-sensitive detection of exo-miRNA-15a-5p. Compared with the non-local reaction mode and the traditional enzyme-free DNA amplification strategy, RHOA was proven to possess more efficient amplification performance. In addition, the catalytic ability analysis of the cascade enzyme system also confirmed that the local space conferred a great performance improvement. The integration of these advantages allowed the ECL biosensor to reach an astonishing LOD value of 1.5 aM, and involved a wide linear concentration ranged spanning 10 orders of magnitude, surpassing most of the reported miRNA biosensing strategies. Furthermore, the developed ECL biosensor possessed excellent specificity, repeatability and stability, and has been successfully applied in the detection of clinical endometrial cancer patient samples, which was highly consistent with the results of qRT-PCR. Therefore, the ECL biosensing strategy is a focused and meaningful attempt to localize reaction, significantly improving the efficiency of enzyme-free amplification and cascaded nanozyme-catalyzed reaction, and provides a reliable approach forward for the further development of exo-miRNA biosensing in the future.

## Supplementary information

**Additional file 1**: **S1**. Exosome extraction. **S2**. miRNA extraction. **S3**. miRNA extraction. **S4**: Rolling circle amplification (RCA). **S5**: Polyacrylamide gel electrophoresis (PAGE). **S6**: quantitative Reverse transcription‑PCR (qRT‑PCR). **Table S1**: Oligonucleotide sequences employed in this work. **Fig. S1**: PAGE characterization of circular DNA Q. **Fig. S2**: ATM characterization of RHOA. **Fig. S3**: Verification of the storage stability of aqueous Cu_2_O solutions. **Fig. S4**: XRD characterization of Fe-Zr MOF. **Fig. S5**: BET analysis of Fe-Zr MOF. **Fig. S6**: Catalytic kinetics of G4 nanozymes with different structures. **Fig. S7**: Optimization of major experimental conditions. **Fig. S8**: Optimization of electrode buffer pH and glucose concentration. **Fig. S9**: Optimization of the concentration of chloroauric acid and the concentration of Cu_2_O/Au drip added to the electrode surface. **Fig. S10**: Verification of the homogeneity of Cu_2_O/Au. **Fig. S11**: Verification of the reproducibility of this biosensor. **Fig. S12**: Characterization of serum exosomes. **Table S2**: Comparison of ECL biosensor with other reported methods for miRNA detection.

## Data Availability

All data generated or analyzed during this study are included in this article and the Additional Information. The additional file is available.
