# Supplementary material for: Dancing in local space: rolling hoop orbital amplification combined with local cascade nanozyme catalytic system to achieve ultra-sensitive detection of exosomal miRNA
Source: J Nanobiotechnology. 2022 Aug 2;20:357. doi: 10.1186/s12951-022-01568-6 (PMC9344616; doi:10.1186/s12951-022-01568-6)
Supplement: Supplementary file 1 — Additional file 1: S1. Exosome extraction. S2. miRNA extraction. S3. miRNA extraction. S4: Rolling circle amplification (RCA). S5: Polyacrylamide gel electrophoresis (PAGE). S6: quantitative Reverse transcription‑PCR (qRT‑PCR). Table S1: Oligonucleotide sequences employed in this work. Fig. S1: PAGE characterization of circular DNA Q. Fig. S2: ATM characterization of RHOA. Fig. S3: Verification of the storage stability of aqueous Cu2O solutions. Fig. S4: XRD characterization of Fe-Zr MOF. Fig. S5: BET analysis of Fe-Zr MOF. Fig. S6: Catalytic kinetics of G4 nanozymes with different structures. Fig. S7: Optimization of major experimental conditions. Fig. S8: Optimization of electrode buffer pH and glucose concentration. Fig. S9: Optimization of the concentration of chloroauric acid and the concentration of Cu2O/Au drip added to the electrode surface. Fig. S10: Verification of the homogeneity of Cu2O/Au. Fig. S11: Verification of the reproducibility of this biosensor. Fig. S12: Characterization of serum exosomes. Table S2: Comparison of ECL biosensor with other reported methods for miRNA detection. [file 12951_2022_1568_MOESM1_ESM.docx]

**Additional file 1**

**Dancing in local space:** **rolling hoop orbital amplification combined with local cascade nanozyme catalytic system to achieve ultra-sensitive detection of exosomal miRNA**

Xin Gao^a,1^, Haiping Wu^a,1^, Yujian Li^b^, Lu Zhang^a^, Mingxuan Song^a^, Xuhuai Fu^c^, Rui Chen^a^, Shijia Ding^a^, Jiawei Zeng ^d,^*^*^*, Jia Li^e^*^,*^*, Ping Liu^a,f*^

^a^ *Key* *Laboratory of Clinical Laboratory Diagnostics (Ministry of Education), College of Laboratory Medicine, Chongqing Medical University, Chongqing 400016, P.R. China*

*^b^ Department of Orthopedics, The First Affiliated Hospital of Chongqing Medical University, Chongqing 400016, P.R. China*

*^c^ Department of Clinical Laboratory, Chongqing University Cancer Hospital & Chongqing Cancer Institute & Chongqing Cancer Hospital, Chongqing 400030, China*

*^d^ Department of Clinical Laboratory, Mianyang Central Hospital, School of Medicine, University of Electronic Science and Technology of China, Mianyang 621000, P.R. China*

*^e^ The Center for Clinical Molecular Medical Detection, The First Affiliated Hospital of Chongqing Medical University, Chongqing 400016, P.R. China*

*^f^ Bioscience (Tianjin) Diagnostic Technology CO., LTD., Tianjin 300399, China*

*Correspondence: [20098181@163.com](mailto:20098181@163.com) (PL), [lijia20210203@163.com](mailto:lijia20210203@163.com)(JL) and [zjweee@sina.cn](mailto:zjweee@sina.cn) (JZ)

Tel: +86-23-68485688; Fax: +86-23-68485786.

^1^ Xin Gao and Haiping Wu contributed equally to this work.

**Experimental**

**S1:** **Exosome extraction.** In this work, the Exosome Isolation Kit (KeyGEN BioTECH, China) was chosen for exosome extraction. The serum samples were centrifuged at 3000 g for 10 min at 4 ℃. Subsequently, the supernatant was collected and filtered with 0.22 µm filter. 1 mL filtered supernatant was transferred to a centrifuge tube, and 10 µL Exosome Isolation Reagent A was added and shaken well. The mixture was reacted at 4 ℃ for 60 min. Then, 250 μL exosome isolation reagent B was added in the above mixture and continued to react at 4 ℃ for 60 min. Finally, the extraction mixture was centrifuged at 12000 g at 4°C for 20 minutes. After discarding the supernatant, the precipitated exosomes were resuspended in 100 μL 1×PBS.

**S2:** **miRNA extraction.** The Serum/Plasma RNA Purification Kit (EZBioscience, USA) was used for exo-miRNA extraction. The exosomes extracted in the previous step were centrifuged with 1 mL, and the supernatant was discarded. Subsequently, 500 μL Lysis Buffer was mixed in the precipitated exosomes, and the exosomes were completely lysed by pipetting about 10 times. Then, 100 μL Buffer A was added to the exosome lysis solution, mixed by inversion, and incubated at room temperature for 3 min. The mixture was centrifuged at 12000 g for 2 min at 4 °C, and then the supernatant was carefully transferred to a new RNase-free 1.5 mL centrifuge tube. 1.6 volumes of 100% ethanol were added to the transferred supernatant. After thoroughly mixing, the mixture was transferred to the Spin Column. Spin Colum was centrifuged at 4000 g for 1 min at 4 °C, and discarded the liquid. Subsequently, 500 μL washing buffer 1 was added to the column and centrifuged at 12000 g at 4 °C for 1 min, and then the liquid was discarded again. Washing buffer 1 was replaced with washing buffer 2 and the same operation was performed again. Then, the washed Spin Column was directly transferred to a 1.5 ml centrifuge tube without RNase, and the lid was opened and kept in the air for 2 min. The center of the column was added with 30 μL Elution Buffer, incubated at room temperature for 2 min, and centrifuged at 12000 g at 4 °C for 1 min. Finally, the eluate was transferred to Spin Column, incubated for 5 minutes and centrifuged again to obtain exosomal miRNA.

**S3: Synthesis of circular DNA.** The circular primer (1 µM) and linear template Q (1 µM) were mixed at a concentration ratio of 1:1, heated at 95 °C for 5 min, and then slowly cooled to room temperature. Subsequently, 10 U T4 DNA ligase (Thermo Scientific, Shanghai, China) was added into the mixture, reacted at 22 ℃ for 2 h and inactivated at 65 ℃ for 10 min. Finally, 10 U Exonuclease I and 10 U Exonuclease III (Thermo Scientific, Shanghai, China) were added to purify circular DNA. The mixture was incubated at 37 ℃ for 50 min and then transferred to 85 ℃ and incubated for 15 min to inactivate the exonuclease.

**S4: Rolling circle amplification (RCA).** In this work, RCA was utilized to verify the synthesis of circular DNA. The detailed experimental steps were as follows: The circular template was selected as the amplification template for RCA. The total volume of the RCA reaction system was 20 µL, which contains 2 µL Q (100 nM), 2 µL circularization template (100 nM), 1 µL phi29 DNA polymerase (10 U), 2 µL dNTP (2 mM), 2 µL 10× phi29 DNA polymerase Buffer (30 mM Tris-acetate (pH 7.9 at 37 °C), 100 mM Mg-acetate, 660 mM K-acetate, 1% (v/v) Tween-20, 10 mM DTT). The reaction mixture was incubated at 37 ℃ for 1 h, and heated at 65 ℃ for 10 min to inactivate the polymerase.

**S5:** **Polyacrylamide gel electrophoresis (PAGE).** Native PAGE was performed on 12 % acrylamide gels in 1×TBE buffer (90 mM Tris-HCl, 90 mM boric acid, 2 mM EDTA, pH 7.9). 10 μL each sample was mixed with 2 μL 12× loading buffer respectively and then loaded into the lane. The gel electrophoresis was carried out for 40 min at a constant voltage of 110 V. The PAGE gel was imaged using a Molecular Imager Gel Doc XR (Bio-Rad) after GelRed staining for 30 min.

**S6:** **quantitative Reverse transcription‑PCR (qRT‑PCR).** The detection samples of qRT-PCR were derived from miRNAs in serum exosomes of patients with endometrial cancer. The detail extraction steps were as above. First, the extracted miRNA was reverse transcribed into cDNA using the miRNA First Strand cDNA Synthesis (tailing reaction) kit (Sangon Inc. Shanghai, China), incubated at 37 °C for 60 min, and then incubated at 85 °C for 5 min. qRT-PCR was performed via the BlasTaqTM 2 × PCR MasterMix Kit (Applied Biological Materials (ABM) Inc. Canada). The detailed PCR cycling conditions were as follows: 95 °C for 3 min, followed by 40 cycles of 95 °C for 15 s and 60 °C for 1 min.

The primers for qRT-PCR analysis were as follows: miRNA-15a-5p forward primer: 5' - AAG CGC CTT AGC AGC ACA TAA T - 3'. U6 forward primer and universal PCR reverse primer used miRNA First Strand cDNA Synthesist (tailing reaction) kit.

**Table** **S1.** **Oligonucleotide sequences employed in this work.**

| Oligonucleotide | Abbreviation | Sequence (5'-3') |
| --- | --- | --- |
| DGH | Catalytic kinetics of G4 nanozymes with different structures | Hemin-CTGATCCTGACTAACTTACC |
| P-G4 |  | ATATGTCGAGGATGCAGGCGGGTAAGTTAGTCAGGATCAGAGGGTGGGTGGGTGGG |
| H-G4 |  | ATATGTCGAGGATGCAGGCGGGTAAGTTAGTCAGGATCAGAGGGTAGGGCGGGTTGGG |
| H_3_G_1_ |  | Phos-TTTTGGGTAGGGCGGGTTGGGA-Hemin |
| H_5_G_1_ |  | Hemin-AGGGTAGGGCGGGTTGGGTTTT-Phos |
| miRNA-15a-5p | Construction of RHOA | TAGCAGCACATAATGGTTTGTG |
| S |  | CACAAACCATTATGTGCTGCTAGGCATTTTTTTCGATGT |
| S’ |  | TCCTTGACTTTCAGCGGATCAAC*rAG*TCCTAGTGAGCTTTTTTAGACGGATGGAG*rAG*ACTATGTGCTCGTTTTTCGATGT |
| L |  | AAGTCAAGGAACATCGAAAA |
| Linear  template Q |  | *Phos*-AGCACATAGTTCCGAGCCGGTCGAACTCC AA CCGTTTTTT*CCTACTAGGATCCGAGCCGGTC GAAGTTGCCTAGC* |
| Cyclization  primer |  | ACTATGTGCTGCTAGGCAAC |
| Ra |  | ATCCGCTGTTTTGTTGTAGGTCCTAACTG  TCCCATCTTTTAGCTCACT |
| Rb |  | ATCCGTCTTTTTGTTGTAGGTCCTAACTGT  CCCATCTTTTCGAGCACA |
| FAM-Ra-BHQ | Verification of the RHOA | FAM-ATCCGCTGTTTTGTTGTAGGTCCTAACTGT CCCATCTTTTAGCTCACT-BHQ |
| FAM-Rb-BHQ |  | FAM-ATCCGTCTTTTTGTTGTAGGTCCTAACTGT CCCATCTTTTCGAGCACA-BHQ |
| T | Assembly of ECL biosensor | Phos-TTTGATGGGACAGTT |
| P |  | AGGACCTACAACTTT-SHC3 |
| CP | Validation of local cascaded nanozymes | CGCCTGCATCCTCGACATATTTTTTT-SHC3 |
| HP |  | ATATGTCGAGGATGCAGGCGTTTTTT-Phos |
| T | Entropy driven reaction | TAGCAGCACATAATGGTTTGTG |
| Q |  | CACAAACCATTATGTGCTGCTAAGGGCCGT  AAGTTAGTTGGAGACGTAGG |
| F |  | CCTACGTCTCCAACTAACTTACGGCCCTTAG  CAGCACATAATGG |
| R |  | CCTACGTCTCCAACTAACTTACGG |
| P |  | TTTTTTTTCCCTTAGCAGCACATAATGG |
| miRNA-21 | Specificity of the ECL biosensor | TAGCTTATCAGACTGATGTTGA |
| miRNA-101 |  | TACAGTACTGTGATAACTGAA |
| miRNA-378 |  | ACTGGACTTGGAGTCAGAAGGC |
| miRNA-155 |  | CTCCTACATATTAGCATTAACA |
| miRNA-122 |  | TGGAGTGTGACAATGGTGTTTG |

**Notes:** The sequences marked in red are G4 sequence. The bases marked in green are the DNA enzyme cleavage site. The italicized part in the Q base design is the MNAzyme 1 sequence, and the underlined part is the MNAzyme 2 sequence.

The CP and HP sequence were designed for verifying the local nanozyme catalysis system. As shown in Fig. 2I, Cu_2_O/Au and Fe-Zr MOF/luminol/G4 was modified with CP and HP respectively. The modification method was the same as that described in the experimental part, except that the modified DNA was different. Then, modified nanozymes was hybridized together to form a localized area.

**
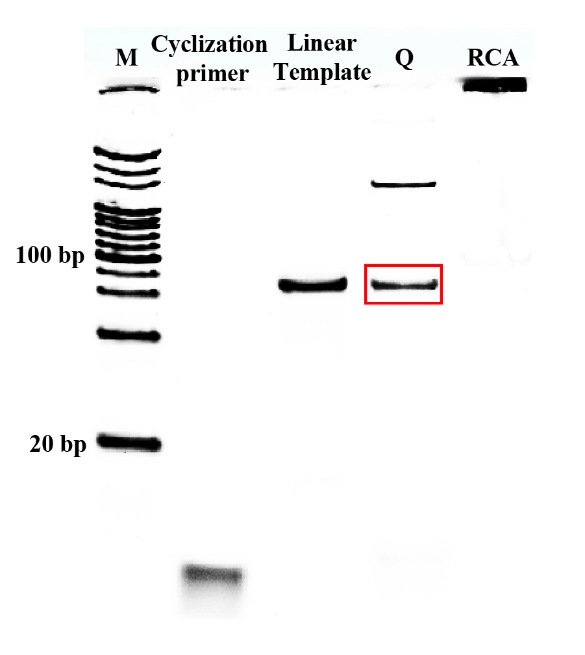
**

**Fig. S1** PAGE characterization of circular DNA Q. The concentration of cyclization primer, linear template and Q was all 1 µM. The band marked by the red box was the circularized Q, because the number of bases in the circularized primer was too few, so the band did not have a significant back shift compared to the linear template. However, the presence of obvious RCA products in lane 5 indicated that the synthesis of Q was successful.


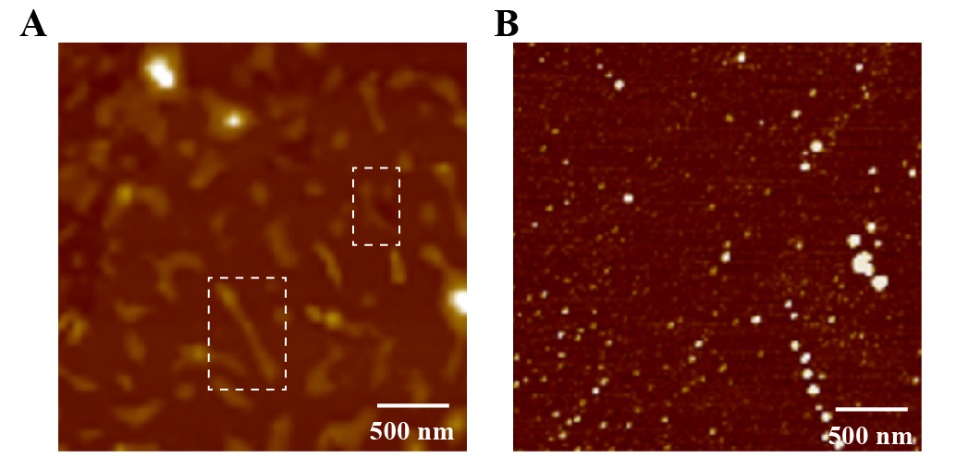


**Fig. S2** ATM characterization of RHOA. A) AFM characterization of the initiation and amplification orbits. Long strips of DNA orbits were visible within the white frame. B) AFM characterization after the completion of the RHOA reaction, it could be seen that the long DNA structures were almost all cleaved.


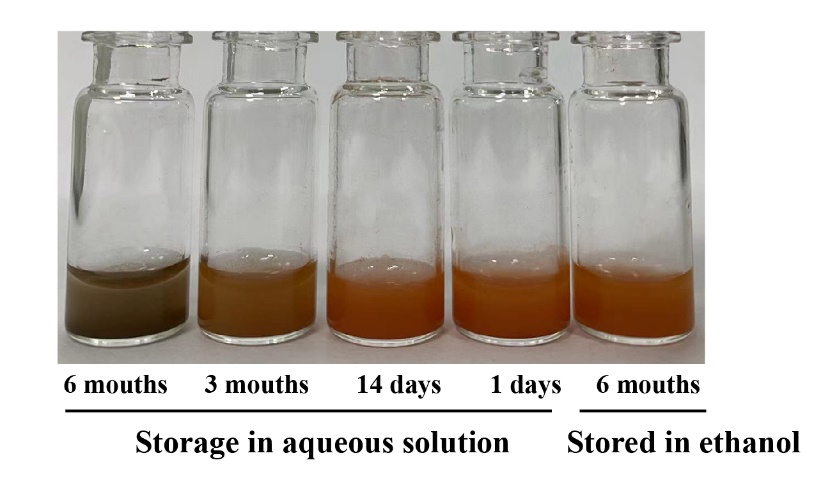


**Fig. S3** Verification of the storage stability of aqueous Cu_2_O solutions.


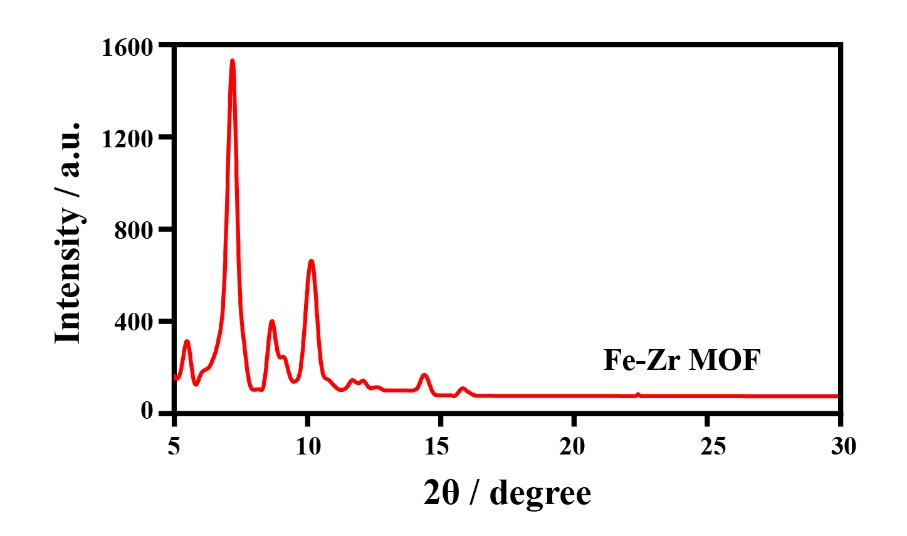


**Fig. S4** XRD characterization of Fe-Zr MOF


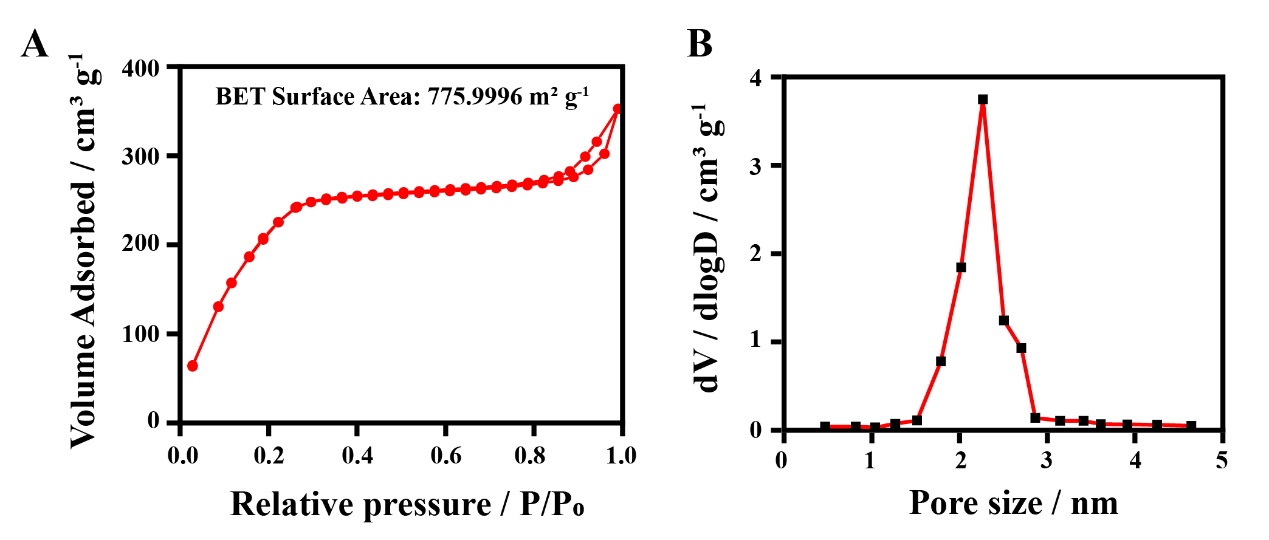


**Fig. S5** BET analysis of Fe-Zr MOF. A) the surface area of Fe-Zr MOF was 775.9996 m^2^ g^-1^. B) the pore size of Fe-Zr MOF was 2.4776 nm.


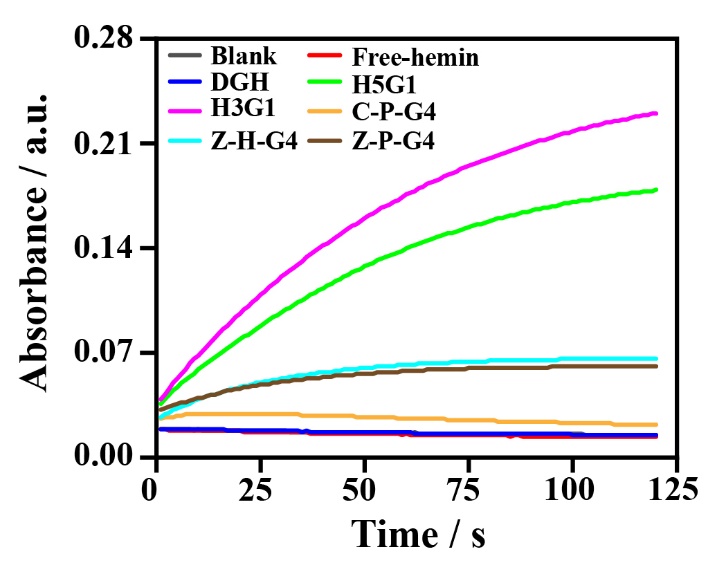


**Fig. S6** Catalytic kinetics of G4 nanozymes with different structures. The results clearly indicated that H3G1 possessed higher catalytic efficiency than other G4 nanozymes, which was selected to applied in this work.


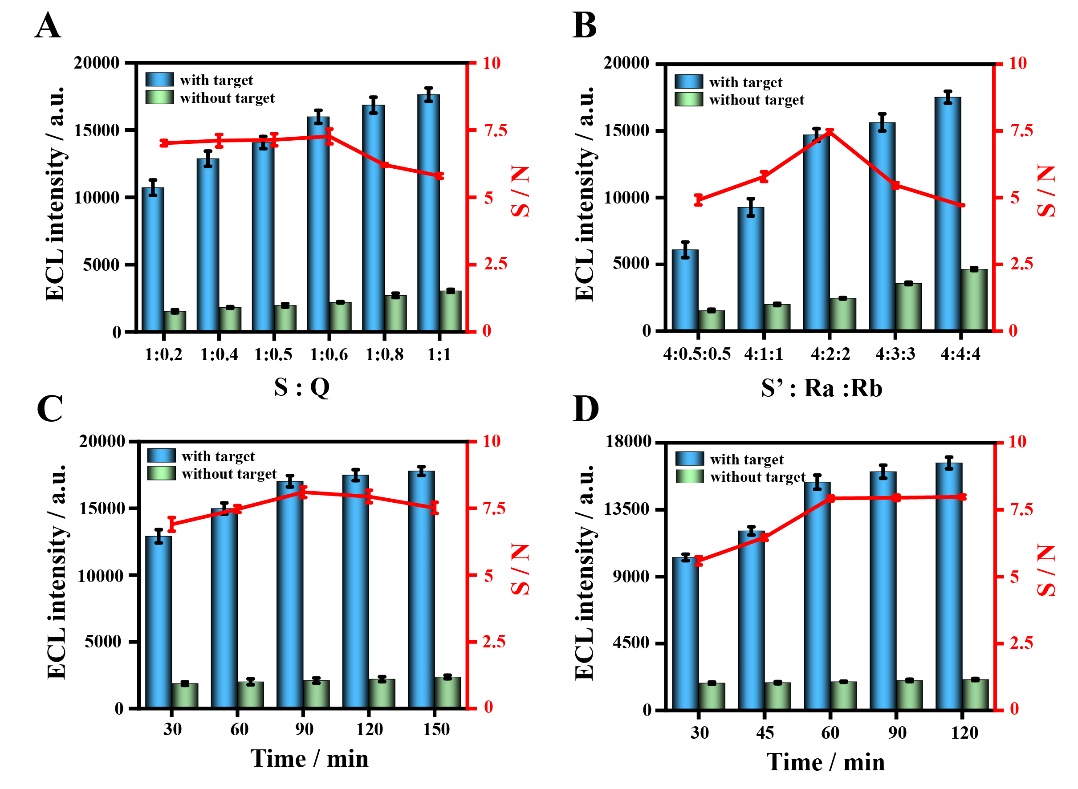


**Fig. S7** Optimization of major experimental conditions: A) the concentration ratio of S and Q. B) the concentration ratio of S’, Ra and Rb. C) the reaction time of RHOA. D) the hybridization time of probes on the electrode surface.


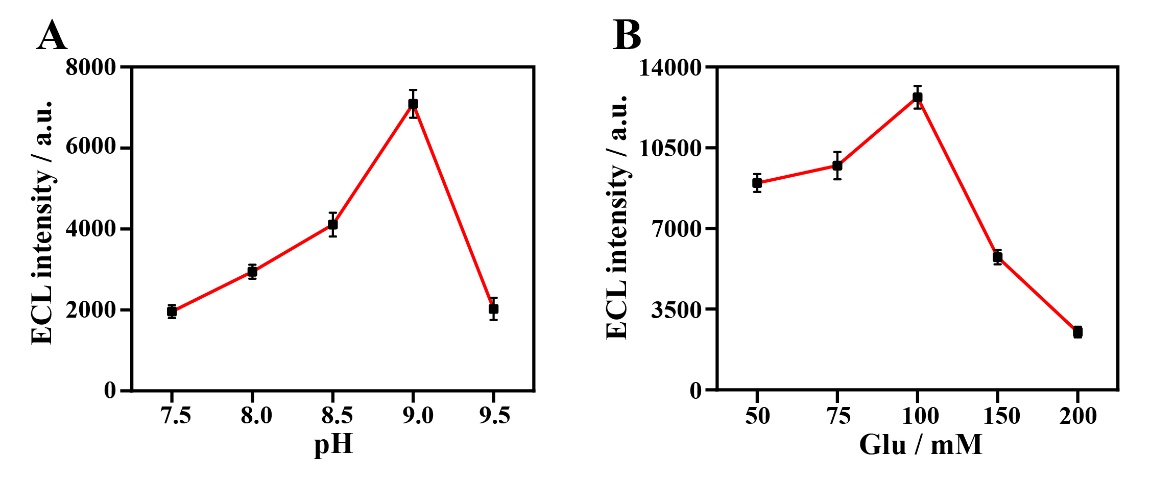


**Fig. S8** Optimization of electrode buffer pH and glucose concentration. A) The maximum signal value was reached when the electrode buffer pH was 9. B) The optimal concentration of glucose was 100 mM.


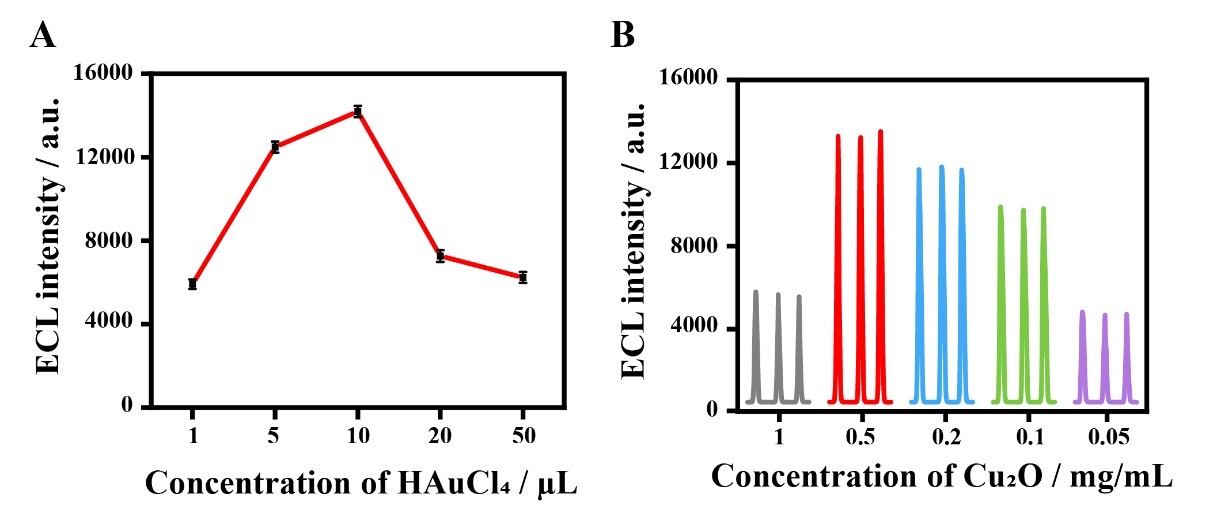


**Fig. S9** Optimization of the concentration of chloroauric acid and the concentration of Cu_2_O/Au drip added to the electrode surface. A) The concentration of chloroauric acid was optimized to obtain the most suitable amount of Au NPs deposited. The optimal in situ reduction condition for Au NPs was the addition of 10 μL of 1% chloroauric acid. B) Optimize the concentration of Cu_2_O/Au dripped on the electrode to achieve the best coating thickness. The ECL signal peaks at a Cu_2_O/Au concentration of 0.5 mg/mL.


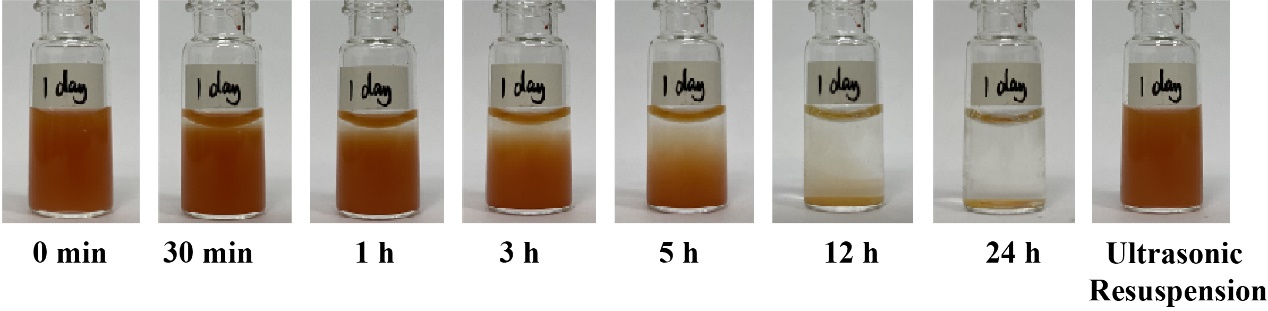


**Fig. S10** Verification of the homogeneity of Cu_2_O/Au. Cu_2_O/Au starts to settle within 30 min and then fully aggregates within 12 h. Dispersion could be restored after resuspension with ultrasound.


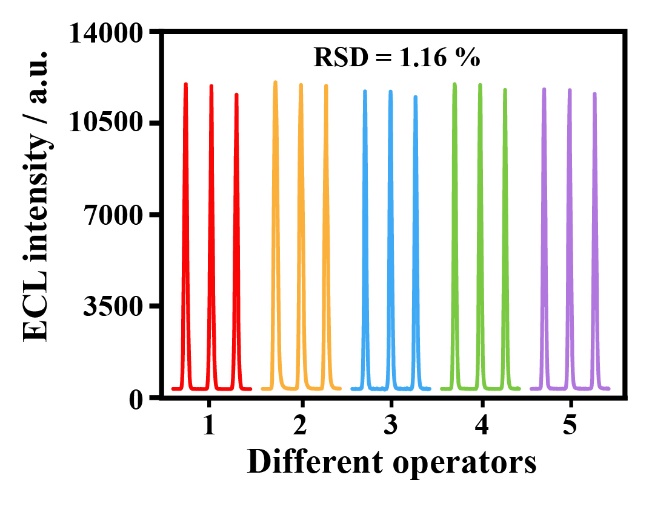


**Fig. S11** Verification of the reproducibility of this biosensor


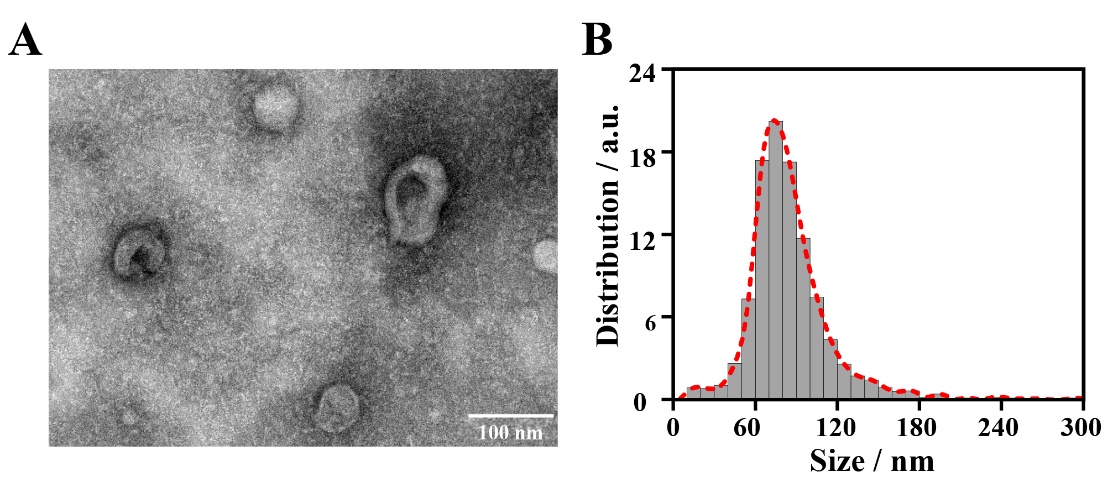


**Fig. S12** Characterization of serum exosomes. A) TEM characterization of exosomes. B) NTA characterization of exosomes: the average diameter of exosomes was 75 nm, which was in line with the classic exosomes size distribution.

**Table S2**. Comparison of ECL biosensor with other reported methods for miRNA detection.

| Detection method | Applied technology | Linear range  (fM) | Detection limit  (aM) | References |
| --- | --- | --- | --- | --- |
| Colorimetry | Cascade enzyme | 2×10^7^-3×10^9^ | 8.34×103 | [1] |
| FL | CRET | 5×10^6^-3.33×10^8^ | 1.7×10^8^ | [2] |
| SPR | HCR | 5×10^-1^-10^4^ | 1.3×10^2^ | [3] |
| EIS | Quantum dots | 10^4^-10^6^ | 5×10^6^ | [4] |
| DPV | Nanozyme | 5×10^2^-10^4^ | 3.3×10^4^ | [5] |
| CL | DNAzyme | 10^3^-5×10^7^ | 3.12×10^7^ | [6] |
| ECL | RHOA and Cascade enzyme | 10^-2^-10^8^ | 1.59 | This work |

**Notes**: FL: Fluorescence. SPR: Surface Plasmon Resonance. EIS: Electrochemical Impedance Spectroscopy. DPV: Differential Pulse Voltammetry. CL: Chemiluminescence. ECL: Electrochemiluminescence. CRET: Chemiluminescence resonance energy transfer. HCR: Hybridization chain reaction. RHOA: Rolling hoop orbital amplification.

**References**

[1] Yu R , Wang R , Wang Z , Liu B , He X , Dai Z . An enzyme cascade sensor with resistance to the inherent intermediate product by logic-controlled peroxidase mimic catalysis. Chem Commun (Camb). 2021;57(16):2089-2092.

[2] Zhang P, Ouyang Y, Willner I. Multiplexed and amplified chemiluminescence resonance energy transfer (CRET) detection of genes and microRNAs using dye-loaded hemin/G-quadruplex-modified UiO-66 metal-organic framework nanoparticles. Chem Sci. 2021;12(13):4810-4818.

[3] Yang CH, Wu TH, Chang CC, Lo HY, Liu HW, Huang NT, Lin CW. Biosensing Amplification by Hybridization Chain Reaction on Phase-Sensitive Surface Plasmon Resonance. Biosensors (Basel). 2021;11(3):75.

[4] Mansuriya BD, Altintas Z. Carbon Dots: Classification, Properties, Synthesis, Characterization, and Applications in Health Care-An Updated Review (2018-2021). Nanomaterials (Basel). 2021;11(10):2525.

[5] Cajigas S, Alzate D, Orozco J. Gold nanoparticle/DNA-based nanobioconjugate for electrochemical detection of Zika virus. Microchim Acta. 2020;187(11):594.

[6] Yang H, Weng B, Liu S, Kang N, Ran J, Deng Z, Wang H, Yang C, Wang F. Acid-improved DNAzyme-based chemiluminescence miRNA assay coupled with enzyme-free concatenated DNA circuit. Biosens Bioelectron. 2022;204:114060.
